# Analyzing cannabinoid-induced abnormal behavior in a zebrafish model

**DOI:** 10.1371/journal.pone.0236606

**Published:** 2020-10-08

**Authors:** Akihiro Hasumi, Hideyuki Maeda, Ken-ichi Yoshida

**Affiliations:** Department of Forensic Medicine, Tokyo Medical University, Shinjyuku-ku, Tokyo, Japan; National Institute of Child Health and Human Development, UNITED STATES

## Abstract

In this study, we investigated locomotor activity and responses to repeated light and dark stimuli to assess cannabinoid-induced abnormal behavior in zebrafish larvae (*Danio rerio*), as an alternative to standard rodent models. To induce the desired responses, we used cannabidiol and WIN55,212–2, two major cannabinoid components. A repeated light and dark test was used to assess how drug exposure influences locomotory responses. Larvae were examined after moderate cannabidiol and WIN55,212–2 exposure and at 24 h after transfer to untreated water. We found that cannabidiol did not produce a dose-dependent inhibitory effect on locomotor activity, with both 0.5 and 10 μg/mL concentrations reducing movement velocity and the total distance moved. However, 10 μg/mL cannabidiol was observed to attenuate the responses of larvae exposed to darkness. No differences were detected between the control and cannabidiol-treated groups after 24 h in fresh water. Fish treated with WIN55,212–2 at 0.5 and 1 μg/mL showed virtually no activity, even in darkness, whereas a concentration of 10 μg/mL induced mortality. A 24-h period in fresh water had the effect of reversing most of the drug-induced immobilization, even in the WIN55,212-2-treated groups. Larvae were also evaluated for their responses to cannabidiol subsequent to an initial exposure to WIN55,212–2, and it was accordingly found that treatment with cannabidiol could attenuate WIN55,212-2-induced abnormal immobilization, whereas equivalent doses of cannabidiol and WIN55,212–2 produced a mixed response. In conclusion, the behavioral effects of the two cannabinoids cannabidiol and WIN55,212–2 appear to be ratio dependent. Furthermore, the repeated light and dark test could serve as a suitable method for assaying drug-induced behavior.

## Introduction

Cannabinoid is a generic term for compounds with a chemical structure derived from *Cannabis sativa* plants [1, 2], the behavioral effects of which have yet to be fully elucidated.

Given the potential adverse effects of cannabis, the constituents that cause these side effects need to be identified. In this regard, several assays have been developed that evaluate locomotor activities, startle latency, behavioral modification, and physiological conditions, including cardiac arrest [3–5]. Typically, rodents are the preferred animal model for such studies, owing to their reliability in behavioral screenings, and predictable physiological responses [6–8]. However, during the past decade, zebrafish (*Danio rerio*) have become increasingly widely used as a model for pharmacological and behavioral research to examine the effects diverse psychotropic drugs [9–13]. Zebrafish are easy to handle and are more cost-effective than rats, and also have certain genetic phenotypes and specific proteins similar to those in humans [14].

With respect to locomotion, three variables are typically evaluated when using zebrafish, namely, the distance moved, movement velocity, and the duration of movement [15–18]. However, monitoring these variables alone would provide insufficient evidence of abnormal behavior (including drug-induced symptoms). When attempting to duplicate pharmacological symptoms, researchers can also concurrently measure the sensitivity of responses using a light or dark preference test [19]. Analyses of the residual effect on behavioral functions after withdrawal of the drugs, and the interactive effects should be included in the study [20, 21]. In both rodent and zebrafish models, typical locomotor activity can be examined using distance moved, movement velocity, and movement duration. Rodent models can be used to evaluate drug concentrations in blood examinations [22]. Therefore, researchers interested in zebrafish as a pharmacological model need to identify appropriate alternatives.

In this study, we evaluated the utility of a repeated light and dark test for screening light sensitivity in addition to assessing typical locomotor activity. For the purposes of the study, we used two representative cannabinoids, namely, cannabidiol (CBD) and R-(+)-[2,3-dihydro-5-methyl-3- [(morpholinyl)methyl]pyrrolo[1,2.3-de]-1,4-benzoxazinyl]-(1-naphthalenyl)methanone mesylate) (WIN55,212–2; WIN). The pharmacological effects of WIN are very similar to those of delta-(9)-tetrahydrocannabinol (THC), which is derived directly from marijuana and activates cannabinoid 1 and 2 receptors [23, 24]. Apart from those findings, however, there have been no studies that have investigated whether CBD-treated groups fully return to their pre-WIN-treated state in zebrafish.

The objective of this study was to analyze cannabinoid-induced behaviors in the acute and withdrawal phases, and to determine how CBD alters the abnormal behaviors induced by WIN. We hypothesized that CBD would attenuate WIN-induced abnormal behaviors. We evaluated the locomotion of zebrafish larvae during acute drug exposure and drug withdrawal, and, given that light sensitivity tests are common in rodent models, we also examined whether repeated light or dark stimuli, as a measure of light sensitivity, would affect drug-induced locomotory responses. The repeated light and dark test was duplicated after drug-treated zebrafish were placed in drug-free water for 24 h. Interactive effects on activity were assessed by administering CBD after exposure to different concentrations of WIN. We believe that the results obtained using these zebrafish assays could provide valuable insights regarding the potential associations between cannabinoids and unexpected abnormal behaviors, which could result in fatal accidents or cause physical dysfunction.

## Materials and methods

In this study, we used a repeated light and dark test to assess drug-induced activity. When evaluating abnormal behavior, more comprehensive insights on the characteristics of abnormal behavior can be gained not only by determining amounts of activity but also by assessing normal behavioral patterns and the changes in these pattern attributable to drug effects. Given that zebrafish tend to be characterized by higher locomotory activity in the dark than in the light, the light and dark test is often used to analyze changes in fish locomotor activity and behavioral patterns due to drug effects [25–27]. Evaluations based exclusively on the parameters of distance moved and movement speed and duration would provide an insufficient assessment of drug effects, and thus to address this deficiency, we also compared behavioral patterns using the repeated light and dark test.

Typically, animal trials investigating pharmacological effects are conducted immediately after drug administration, or after replacing the drug solution with fresh water. Accordingly, in the present study, we also examined drug-treated fish after a 24-h exposure to fresh unmedicated water. To facilitate analysis of the behavioral responses of treated zebrafish, we used a high-throughput tracking system (Danio Vision XT) and behavioral analysis software (Ethovision XT 11.5) [28].

The Danio Vision XT system is designed to study the movement of small organisms and can track up to 96 individuals simultaneously. The procedure adopted when using this device was as follows. After transferring the larvae from Petri dishes to experimental microtiter plate, the plate is placed in a chamber that can be illuminated with bright lights (i.e., light-on periods) or infrared lights (i.e., darkness or light-off periods) using the associated software. The light intensity used during the experiments was 700 lx, whereas under infrared illumination, the intensity was 0 lx. During the periods in which measurements were obtained, the light stimulus was turned on and off at 15-min interval (i.e., 15 min of bright light followed by 15 min of darkness), and the changes in movement patterns were repeatedly analyzed over six alternating periods of 15-min light and 15-min dark stimuli (i.e., a total measurement time of 3h).

### Fish and fish culture

All animal experiments performed in the present study were approved by the Experimental Animal Committee of Tokyo Medical University (approval number: H30-0020, R1-0119). Healthy, adult zebrafish (*Danio rerio*; wild-type, purchased from Kamihata Fish Industries Ltd., Tokyo, Japan) were housed and raised in aerated breeding units at a density of 10 fish per liter, in water from a recirculating water system supplied with dechlorinated municipal tap water. The fish were maintained under conditions of pH 7.5–8.0, a conductivity of 300–500 μS/cm, a temperature of 26–28°C, and a 14:10 h light:dark photoperiod, and were fed twice daily with flake food. To obtain embryos for the purposes of the present study, male and female zebrafish were paired in the evening, and fertilized embryos were collected from the mated zebrafish and placed in Petri dishes containing fresh water. These embryos were transferred to a 28°C incubator under a 14:10-h light:dark cycle until 4–6 days post fertilization (4–6 dpf), During this time, the embryos were screened to assess overall health and dead embryos were removed daily.

### Chemicals

Although CBD, one of the major compounds present in the marijuana plants (*C*. *sativa*), has certain medicinal properties, its mechanisms of action have yet to be sufficiently established [29, 30]. In the present study, we used CBD diluted with 0.05% methanol and sterile saline (Cayman Chemical, Ann Arbor, MI). WIN (obtained from Adooq Bioscience, Irvine, CA) is a synthetic agent with a high affinity for cannabinoid 1 and 2 receptors, two mixed cannabinoid receptor agonists [31], and was used diluted with 0.05% dimethyl sulfoxide (Dojindo Laboratories, Japan) and sterile saline.

#### 1. Treatment in the acute phase

Zebrafish were exposed to different concentrations of CBD (0.5, 1, 5, and 10 μg/mL) or WIN (0.5, 1, 5, and 10 μg/mL). After 30 min, without replacing the water, use a plastic dropper on the round 96-wells microtiter plate (IWAKI Co., Ltd., Tokyo, Japan) for measurement, which was filled with 300 μL of culture water or dosing water. The animals were replaced (n = 96) and locomotor activity was measured when exposed to the drug.

#### 2. 24 hours after drug withdrawal

Six petri dishes filled with non-treated fresh water were prepared, and a seal was attached to each Petri dish according to concentration. At the end of the acute phase of the experiment, all the fish were transferred from the 96-well microtiter plate to a Petri dish filled with untreated water using a plastic dropper. Petri dishes were divided according to concentration groups, and all Petri dishes were allowed to stand in the breeding environment for 24 h. Thereafter, in the absence of the drug, the fish were again divided into each concentration group, transferred individually to a microtiter plate, and re-measured using the same procedure and equipment used for the acute period administration.

#### 3. Mixed treatment of CBD and WIN

Zebrafish were pre-exposed to CBD (0.5, 1, 5, and 10 μg/mL) in a Petri dish for 30 min, and then WIN (0.5 and 1 μg/mL) was added to each CBD preparation without water replacement. The Petri dish was dosed and mixed. Finally, zebrafish in a Petri dish by CBD+WIN (0.5 + 0.5, 1 + 0.5, 5 + 0.5, 10 + 0.5 μg/mL) or CBD + WIN (0.5 + 1, 1 + 1, 5 + 1, 10 + 1 μg/mL). After a further 30 min, the water was not replaced and the animals were replaced individually in the microtiter plate for measurement, and measured using the same procedure and equipment as used when exposed to the drug.

### Experimental procedure

Zebrafish larvae (4–5 dpf) were maintained in 96-well microtiter plates (1 larva/well) filled with 300 μL of E3 medium (n = 96). Locomotor activity was assessed after CBD and WIN treatments at concentrations of 0.5, 1, 5, and 10 μg/mL (16 larvae/group) were added directly into the wells. CBD+WIN treatments of 0.5+0.5, 1+0.5, 5+0.5, and 10+0.5 μg/mL (16 larvae/group), and CBD+WIN treatments of 0.5+1, 1+1, 5+1, and 10+1 μg/mL (16 larvae/group) were also assessed (n = 384).

The cannabinoid concentrations used in the present study were selected based on those used previously by Connors et al. [32]. In addition, owing to the format of the experiment, it was necessary to use equal numbers of larvae before and after the acute period and after 24 hours of withdrawal in order to obtain valid comparisons.

Fish in each treatment group were then subjected to repeated light and dark stimuli to determine differences in locomotor activities and responses, examined under the alternating 15-min intervals of light and dark. In instances where more than half the fish in the groups had died prior to commencing observations, we omitted the results of reactions in treated groups.

We conducted pre-experimental trials and established that zebrafish larvae displayed a hyperactive response for a few seconds following a sudden light stimulus, and immediately thereafter showed moderate amounts of movement in the light. In contrast, when exposed to a sudden dark stimulus, zebrafish larvae displayed a hyperactive response for between 5 and 15 min thereafter, after which there was a gradual reduction in movement. In this regard, it is predicted that the results obtained for the first and second exposures to repeated light stimuli would differ considerably from those of control larvae, whereas the responses to the third to sixth exposures would becoming increasing similar to those of the control larvae due to fatigue or acclimation. The findings of our previous study indicated that the interval between light and dark states should be within 15 min of measurement to avoid acclimation of the response to these stimuli.

### Statistical analysis

One-way ANOVAs were used for statistical comparisons of the recorded observational data, followed by pairwise post hoc comparisons using Dunnett's test or the Tukey–Kramer test.

Data are presented as the means ± SEMs (standard error of the mean). Statistical analysis was performed using GraphPad Prism 6 for Windows version 6.05. The data were analyzed using Dunnett's test, the Tukey–Kramer test, or Student's t-test at a confidence level of 95%.

## Results

### Effects of different concentrations of CBD during drug exposure and 24-h drug withdrawal

**Fig 1:**
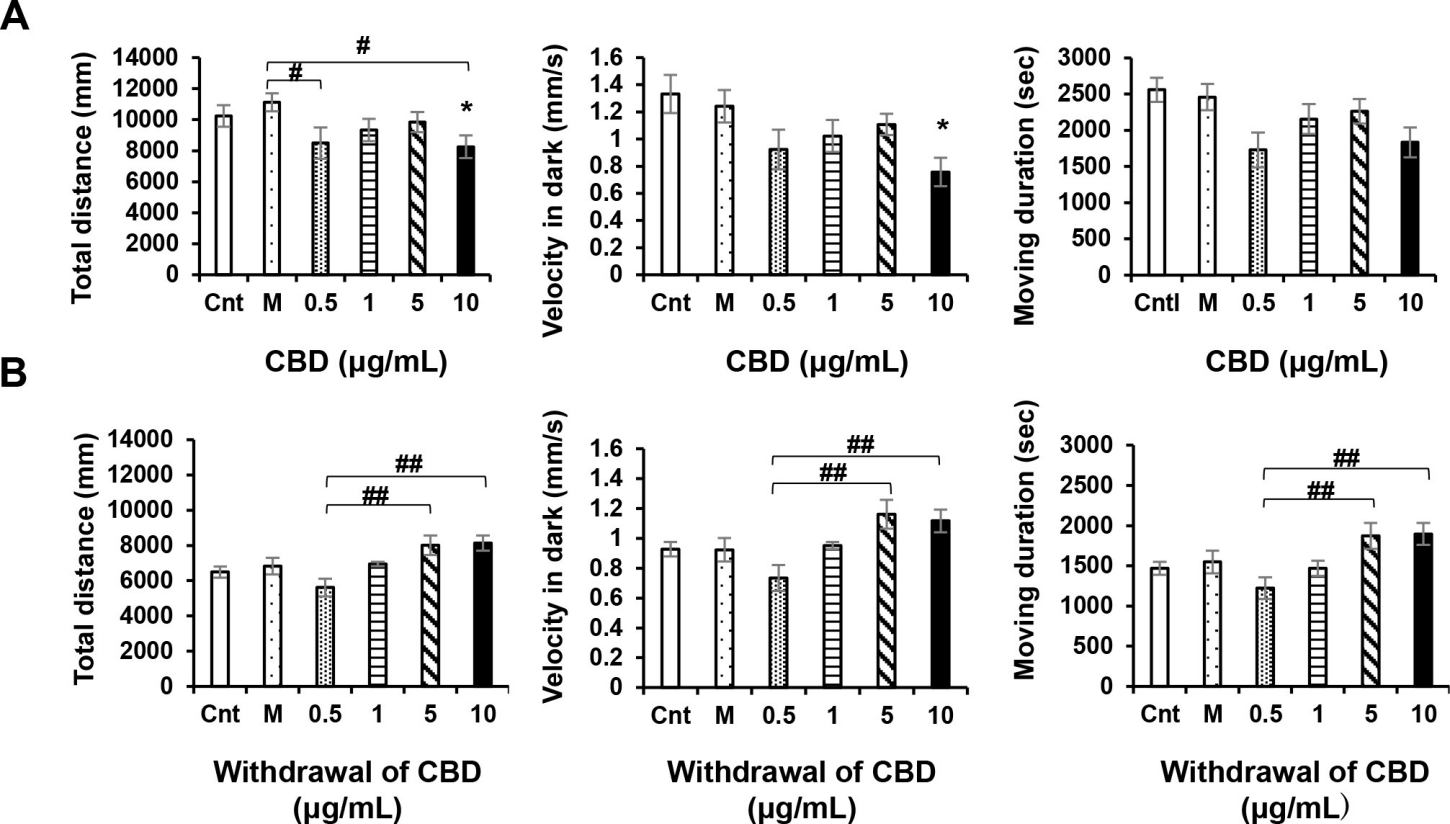
Locomotor effects of cannabidiol (CBD) at different concentrations. After a 24-h withdrawal, CBD (10 μg/mL) induced significant reductions in locomotory activity (A). CBD (5 and 10 μg/mL) induced increases in locomotory activity compared with a dose of 0.5 μg/mL (B). Cnt: control, M: methanol. *P < 0.05, **P < 0.01 vs. control, ^#^P < 0.05, ^##^P < 0.01 vs. drug treatment. Tukey’s multiple comparison test. All values are expressed as the mean ± SEM. In the graphs, the durations of movement are expressed as an absolute time (s). In term of percentages of the total assay time, times of 1000, 2000, and 3000 s correspond to 9.259%, 18.518%, and 27.777%, respectively. Each experiment; n = 96.

**Fig 2:**
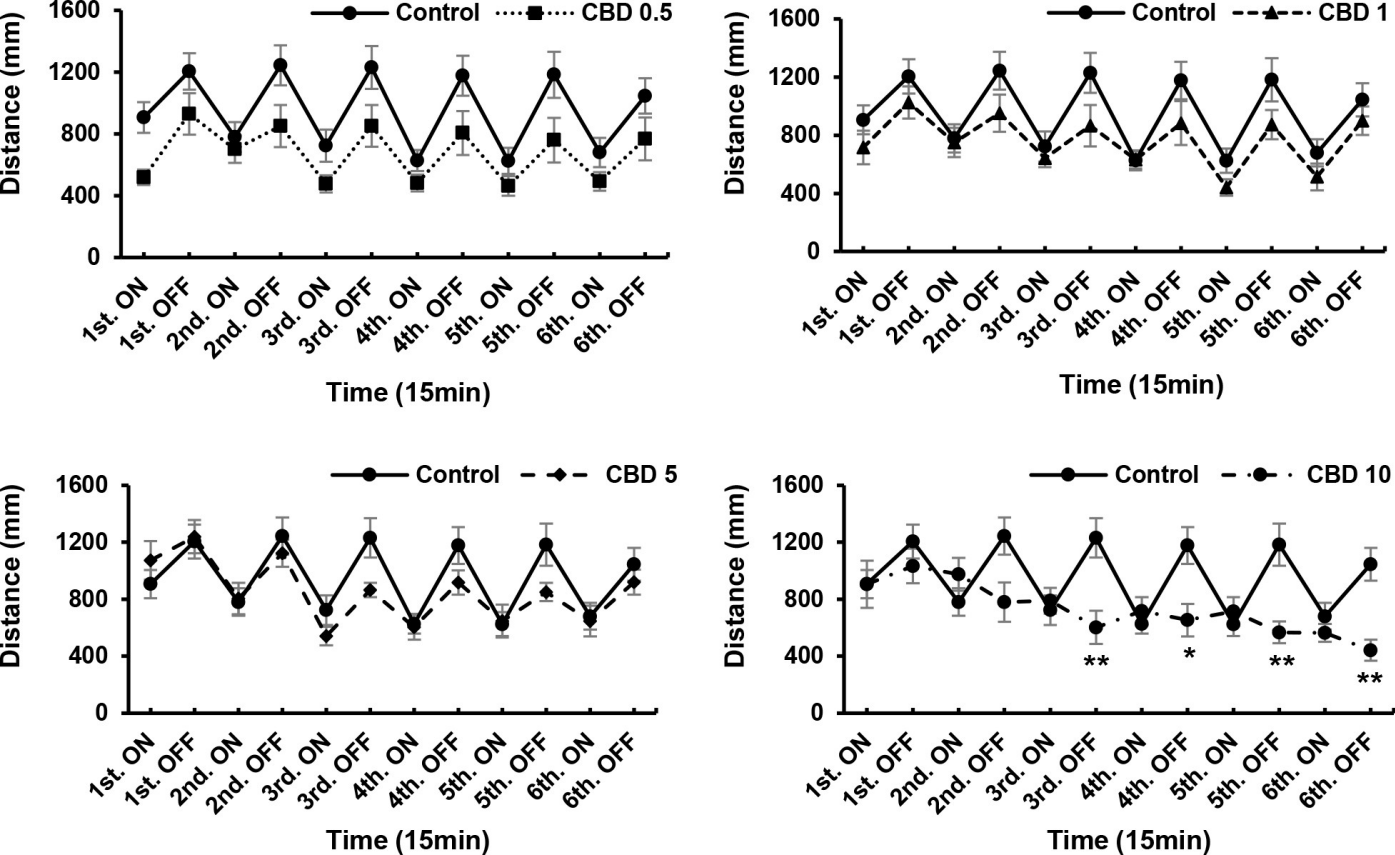
Responses to different concentrations of cannabidiol (CBD) during drug. CBD (10 μg/mL) exerted the strongest effect (i.e., was associated with the weakest responses to darkness) compared with the control. *P < 0.05, **P < 0.01 vs. control, ^#^P < 0.05, ^##^P < 0.01 vs. drug treatment. T-test. All values are expressed as mean ± SEM. Each experiment; n = 96.

**Fig 3:**
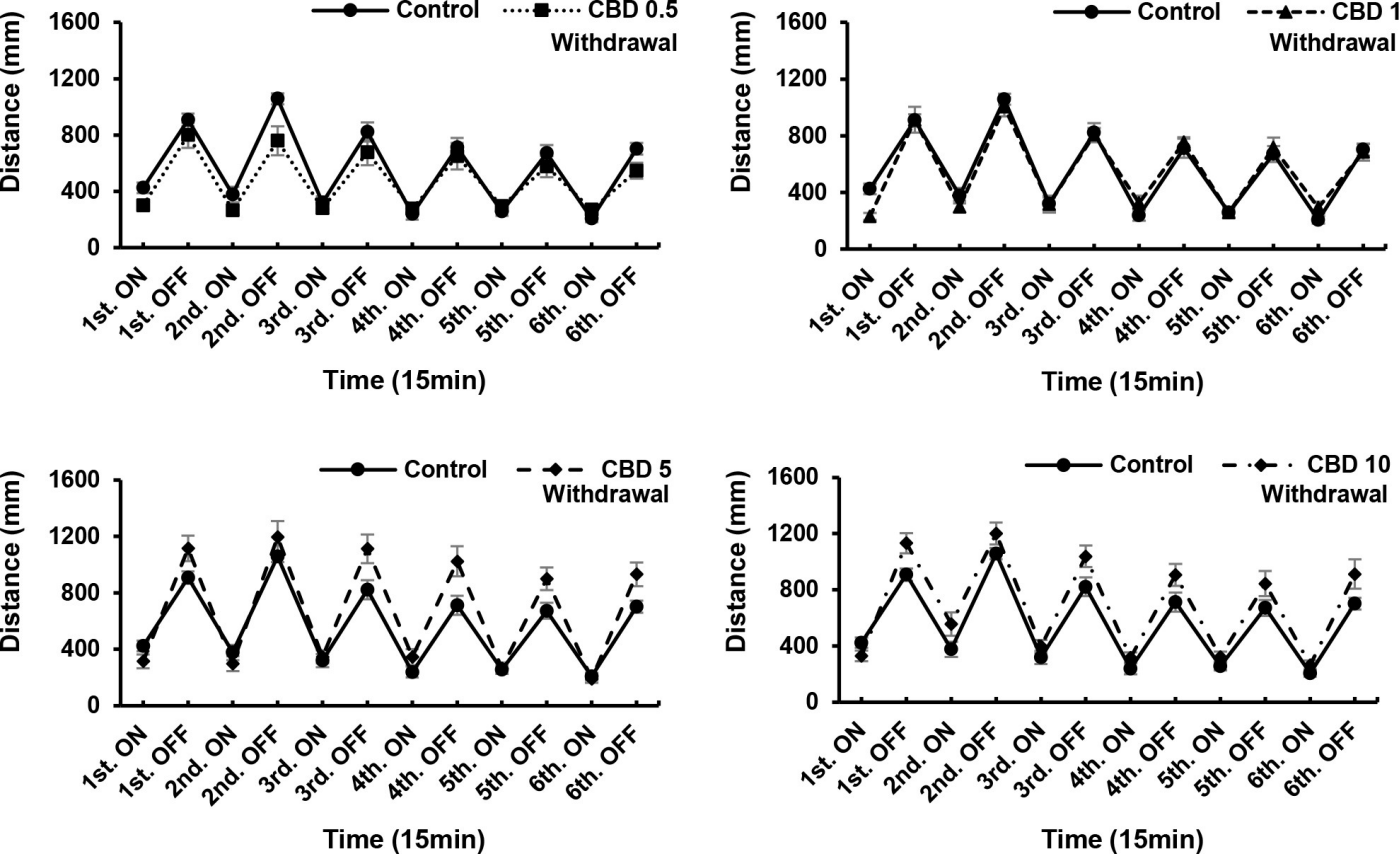
Responses to different concentrations of cannabidiol (CBD) after a 24-h withdrawal. In larvae treated with 10 μg/mL CBD, locomotory responses recovered after a 24-h withdrawal. *P < 0.05, **P < 0.01 vs. control, ^#^P < 0.05, ^##^P < 0.01 vs. drug treatment. T-test. All values are expressed as mean ± SEM. Each experiment; n = 96.

ANOVA revealed significant CBD treatment effects [F (5, 90) = 3.169, P = 0.0111]; CBD withdrawal effects [F (5, 90) = 4.818, P = 0.0006]. CBD at a concentration of 10 μg/mL significantly influenced the locomotor activity of zebrafish larvae (P < 0.05; Fig 1A), and induced reductions in the total distance moved and velocity of movement. At concentrations of 5 and 10 μg/mL, CDB induced increases in locomotor activity compared with 0.5 μg/mL CBD. However, after 24 h in the absence of the drug, we detected no significant differences compared with the control. We found that responses to 0.5 μg/mL CBD differed significantly from those to 5 and 10 μg/mL, which can be attributed to the fact that locomotor activity, including that of control group larvae, tended to decrease during the 24-h withdrawal period, although the motor activity of larvae exposed to high concentrations of CBD (5 and 10 μg/mL) did not show a significant reduction. Therefore, there were difference in the motor activity of the 5 and 10 μg/mL CBD groups compared with the 0.5 μg/mL CBD treatment, in which the larvae initially showed a reduction motor activity (Fig 1B). A CBD concentration of 10 μg/mL was found to have the strongest effect (weak responses to darkness) compared with the control (P < 0.01; Fig 2), with the weakest responses occurring at intervals three, five, and six. However, we detected no significant differences in the locomotory responses of the remaining drug-treated groups. Nevertheless, after a 24-h withdrawal, we observed that even in larvae exposed to 10 μg/mL CBD, the locomotory responses recovered (Fig 3). In the acute trials, the responses tended to decrease both in the light and the dark, although with the exception of the 10 μg/mL group, the recorded differences were not significant. After a 24-h withdrawal, the responses of fish exposed to 0.5 and 1 μg/mL CBD had almost recovered (Fig 3), whereas in contrast, responses at CBD concentrations of 5 and 10 μg/mL increased overall, albeit non-significantly (Fig 3).

### Effects of different concentrations of WIN during drug exposure and 24-h withdrawal

**Fig 4:**
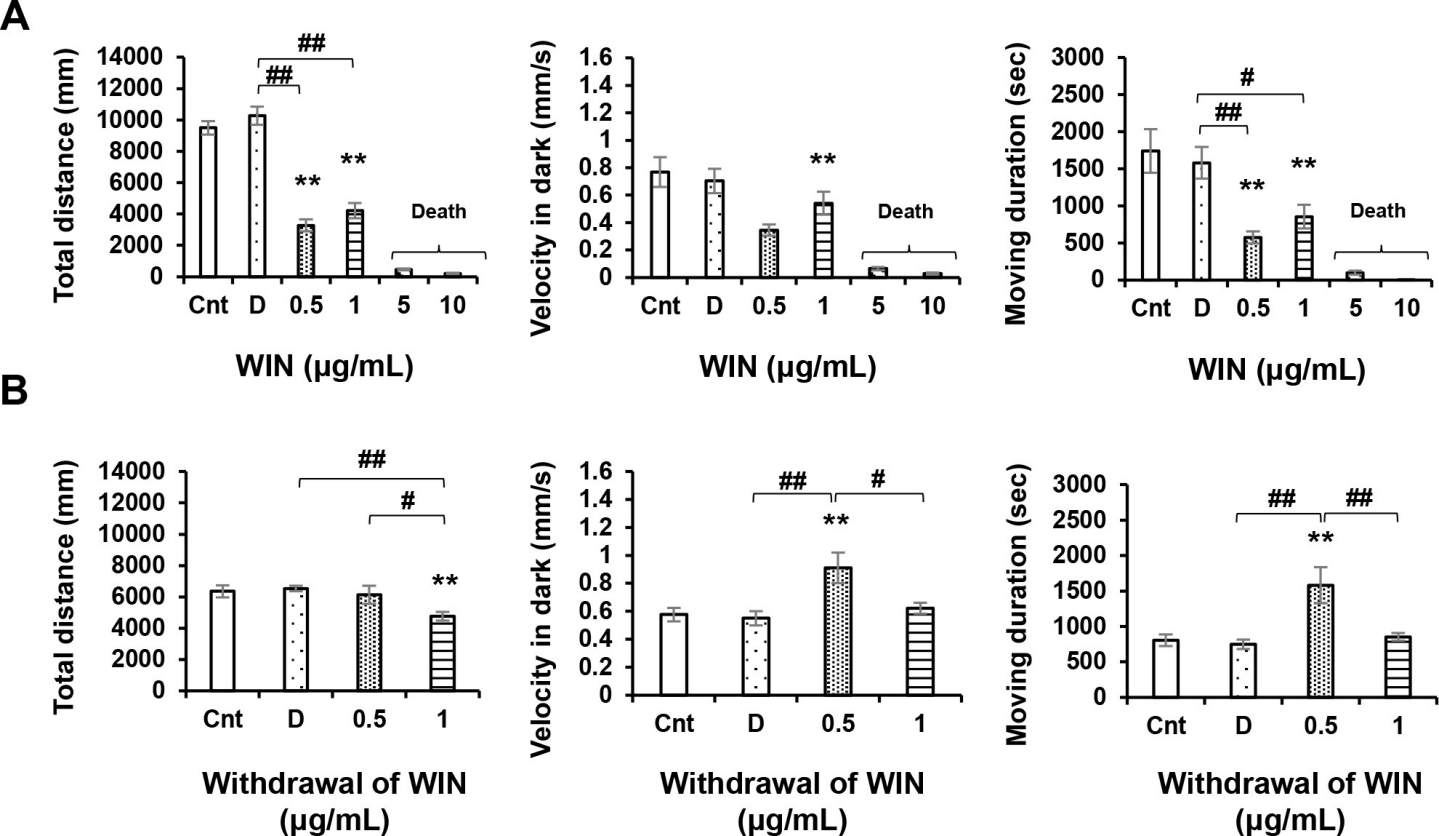
Locomotor effects of WIN55,212–2 (WIN) at different concentrations. After 24 h withdrawal. WIN (0.5 and 1 μg/mL) induced significant reductions in locomotory activity (A). WIN (0.5 μg/mL) induced increases in locomotory activity after 24-h withdrawal (B). Cnt: control, D: dimethyl sulfoxide. *P < 0.05, **P < 0.01 vs. control, ^#^P < 0.05, ^##^P < 0.01 vs. drug treatment. Tukey’s multiple comparison test. All values are expressed as the mean ± SEM. In the graphs, the durations of movement are expressed as an absolute time (s). In term of percentages of the total assay time, times of 1000, 2000, and 3000 s correspond to 9.259%, 18.518%, and 27.777%, respectively. Each experiment; n = 96.

**Fig 5:**
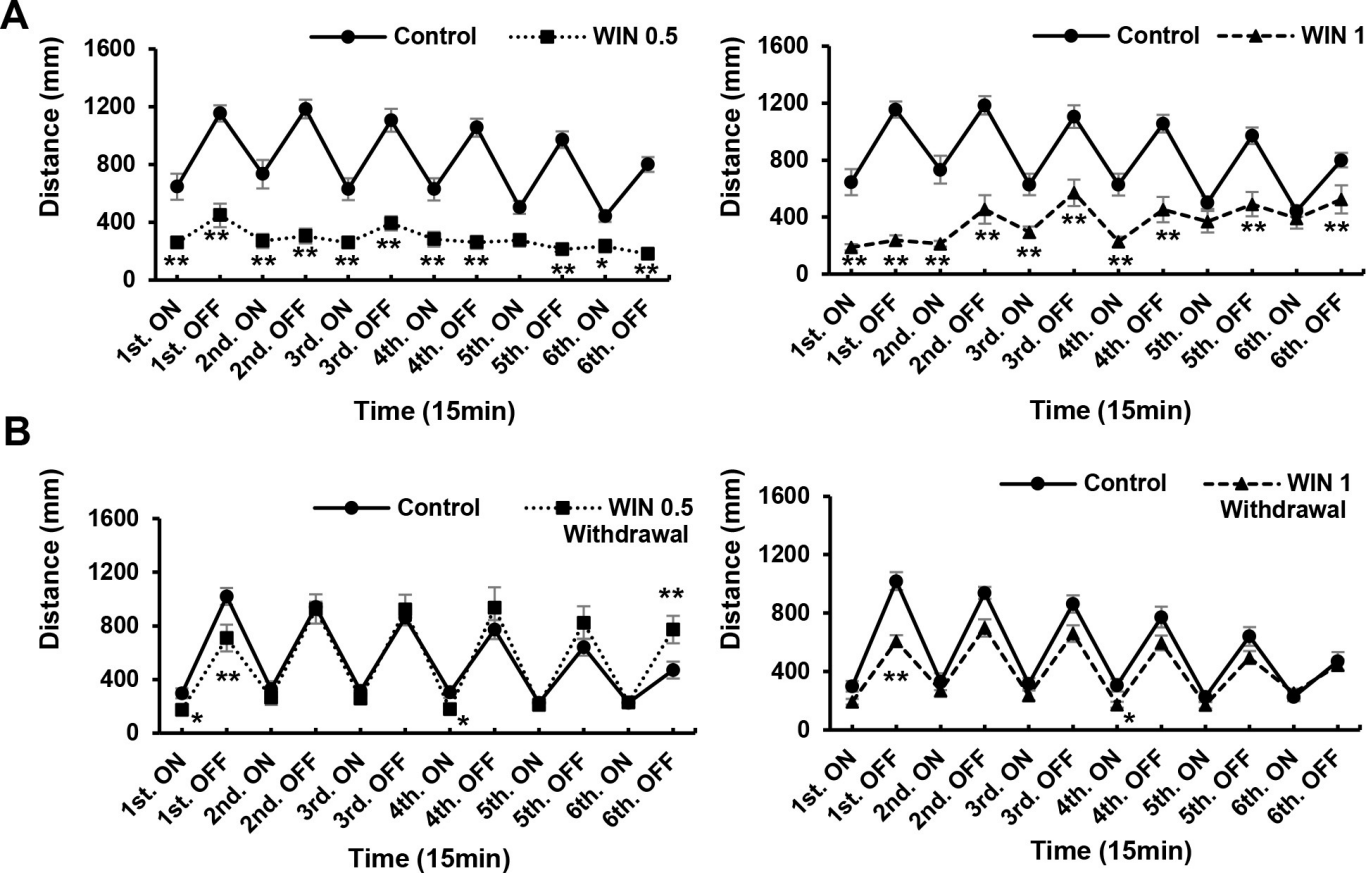
Responses to different concentrations of WIN55,212–2 (WIN) during drug exposure and after a 24-h withdrawal. WIN (0.5 and 1 μg/mL) induced significant reductions in the response to both light and dark stimuli (A). After 24 h in the absence of the drug, at 0.5 μg/mL, there was low activity in the first interval of both light and dark, and in the fourth interval of light. However, increases in responses were observed during the sixth dark interval (B). At a WIN concentration of 1 μg/mL, there was a significant attenuation of responses in the first and fourth light and dark intervals (B). *P < 0.05, **P < 0.01 vs. control, ^#^P < 0.05, ^##^P < 0.01 vs. drug treatment. t-test. All values are expressed as mean ± SEM. Each experiment; n = 96.

ANOVA revealed significant WIN treatment effects [F (5, 89) = 119.8, P = 0.0001(p < 0.05)]; WIN withdrawal effects [F (3, 60) = 4.409, P = 0.0072]. At concentrations of 0.5, and 1 μg/mL, WIN significantly influenced locomotory responses (P < 0.01; Fig 4A), reducing locomotor activity and causing weakened responses to repeated light and dark stimuli (Fig 5A). However, WIN concentrations of 5 and 10 μg/mL proved lethal to zebrafish larvae within less than 24 h (Table 1), with over half of the fish in the 5 μg/mL group and all of the fish in the 10 μg/mL group being killed within this time period. All fish were still alive at the end of the acute trials. Thus, the results obtained for both the 5 and 10 μg/mL groups were omitted from statistical analysis. Concentrations of 0.5 and 1 μg/mL induced increases in locomotor activity after a 24-h withdrawal (P < 0.01; Fig 4B), and the response to light recovered in those larvae exposed to 0.5 μg/mL WIN. In response to both light and dark stimuli, low activity remained during the first and fourth light intervals. However, we observed an increased response during the sixth dark interval (P < 0.01; Fig 5B). Attenuation of both responses in the first and fourth intervals remained significant, whereas the responses of larvae exposed to a WIN concentration of 1 μg/mL were comparable to those of the control larvae during the sixth interval (P < 0.01; Fig 5B).

**Table 1:** Lethal effects of drugs (each experiment; n = 96).

Lethal effects of drugs			
Drug	Concentration	Percentagenumber	Lethal number/Total
WIN	5 (μg/mL)	60%	10 / n = 16
	10 (μg/mL)	100%	16 / n = 16
WIN+CBD (W+C)	W5+C5 (μg/mL)	50%	8 / n = 16
	W10+C10 (μg/mL)	100%	16 / n = 16

WIN55,212–2 (WIN) doses of 5 and 10 μg/mL were lethal at < 24 h. Over half of the fish receiving 5 μg/mL were dead, and all fish receiving 10 μg/mL died within 24 h. However, all zebrafish were still alive at the end of the acute trial.

### Interactions between different concentrations of CBD and WIN during drug exposure

**Fig 6:**
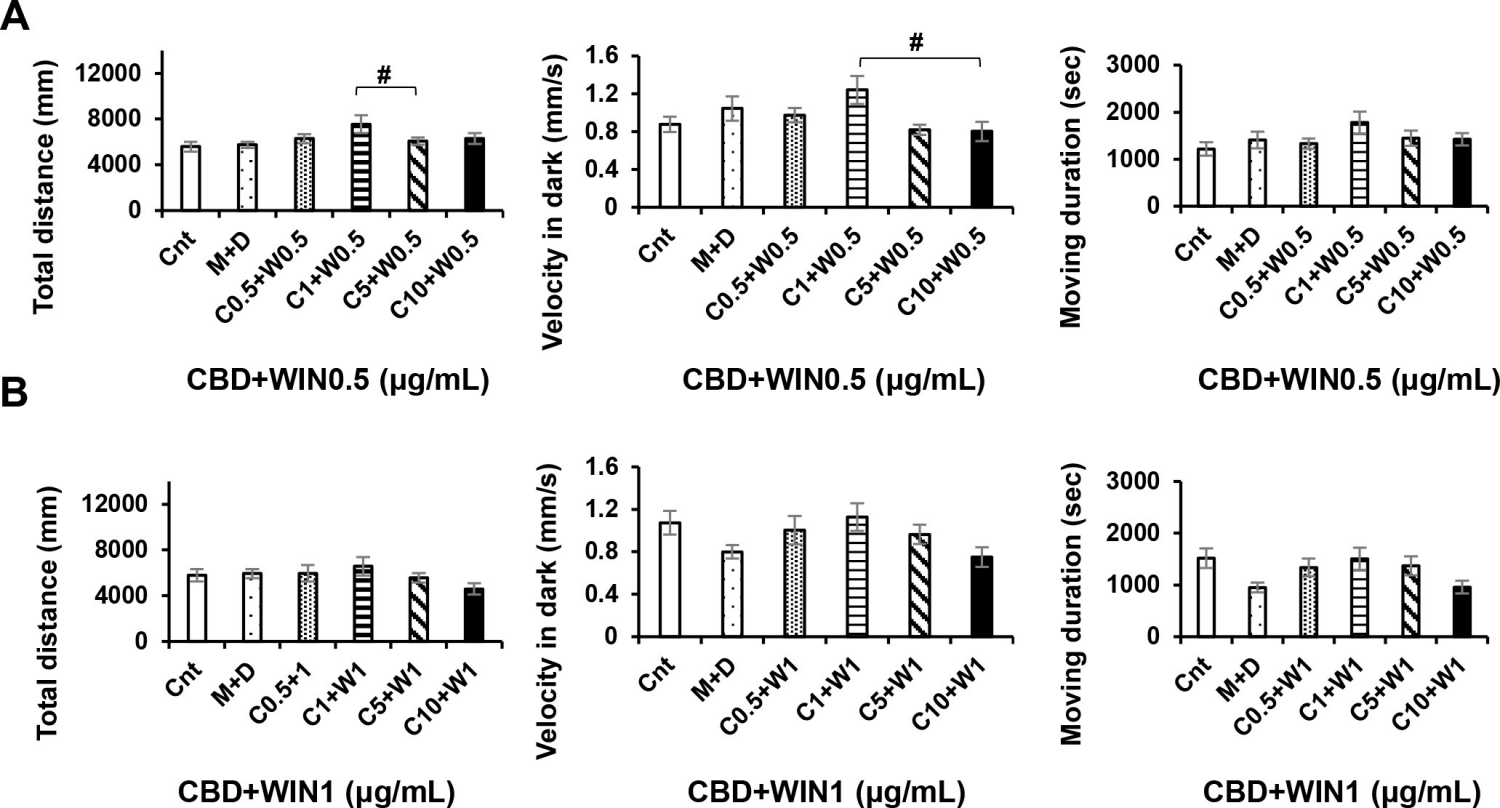
Locomotor effects of CBD+WIN at different concentrations. CBD1 + WIN0.5 induced significant differences in locomotory activity compared with CBD5 + WIN0.5 (A), and CBD10 + WIN0.5 (A). Cnt: control, M: methanol, D: dimethyl sulfoxide. *P < 0.05, **P < 0.01 vs. control, ^#^P < 0.05, ^##^P < 0.01 vs. drug treatment. Tukey’s multiple comparison test. All values are expressed as the mean ± SEM. In the graphs, the durations of movement are expressed as an absolute time (s). In term of percentages of the total assay time, times of 1000, 2000, and 3000 s correspond to 9.259%, 18.518%, and 27.777%, respectively. Each experiment; n = 96.

**Fig 7:**
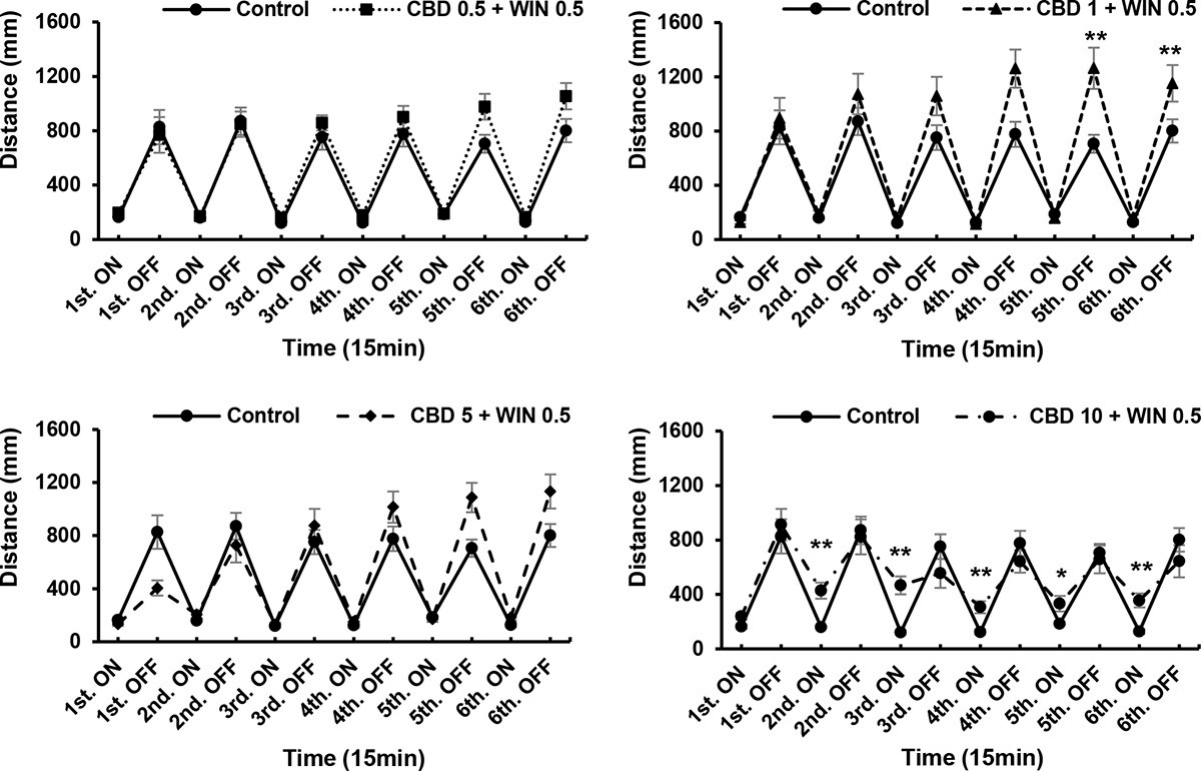
Responses to interactions between different concentrations of cannabidiol (CBD) and WIN55,212–2 (WIN) during drug exposure. Interactions between WIN (0.5 μg/mL) and CBD (0.5, 1, 5, and 10 μg/mL) altered the locomotory responses of zebrafish larvae. Interactions of CBD1 + WIN0.5 (μg/mL) induced significant increases in activity during the fifth and sixth dark intervals, whereas CBD10 + WIN0.5 (μg/mL) induced decreases from the second to sixth light intervals. *P < 0.05, **P < 0.01 vs. control, ^#^P < 0.05, ^##^P < 0.01 vs. drug treatment. T-test. All values are expressed as the means ± SEM. Each experiment; n = 96.

**Fig 8:**
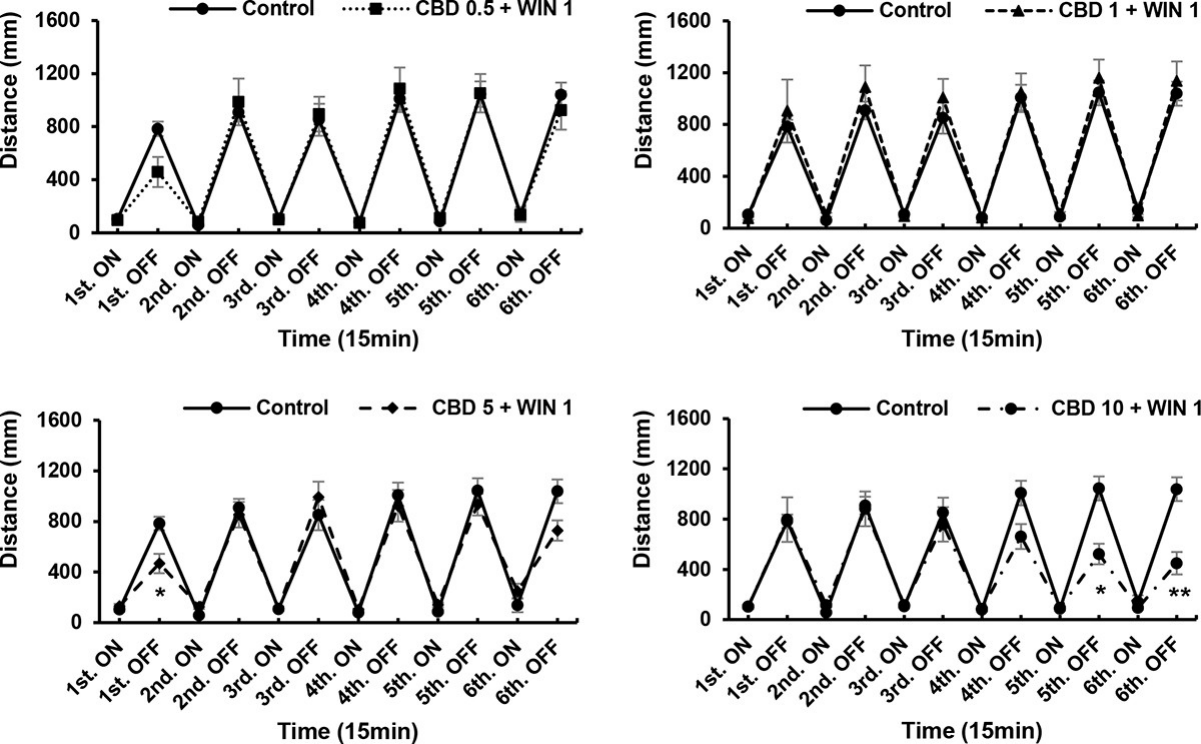
Responses to interactions between different concentrations of cannabidiol (CBD) and WIN55,212–2 (WIN) during drug exposure. Interactions between WIN (1 μg/mL) and CBD (0.5, 1, 5, and 10 μg/mL) altered the locomotory responses of zebrafish larvae. Interactions of CBD5 + WIN1 (μg/mL) induced decreases during the first dark interval, whereas CBD10 + WIN1 (μg/mL) induced decreases in the fifth and sixth dark intervals. *P < 0.05, **P < 0.01 vs. control, ^#^P < 0.05, ^##^P < 0.01 vs. drug treatment. T-test. All values are expressed as the means ± SEM. Each experiment; n = 96.

ANOVA revealed no significant CBD+WIN 0.5 effects [F (5, 90) = 1.949, P = 0.0940]; or CBD+WIN 0.5 effects [F (5, 90) = 1.265, P = 0.2860], whereas Turkey’s test revealed significant CBD1, and 5+WIN 0.5 effects. We found that interactions between WIN (0.5 and 1 μg/mL), and CBD (0.5, 1, 5, and 10 μg/mL) altered the locomotory responses of exposed zebrafish larvae. At concentrations of 0.5, 1, 5, and 10 μg/mL, CBD attenuated the low activity (P < 0.05; Fig 6A and 6B), and weakened the responses induced by WIN (P < 0.01; Figs 7 and 8). Thus, CBD induced increases in locomotor activity and responses to repeated light and dark stimuli in larvae previously exposed to 0.5 and 1 μg/mL WIN. Responses increased during the fifth and sixth dark intervals, when 1 μg/mL CBD (a double dose of CBD 0.5 μg/mL) was administered to larvae pre-treated WIN 0.5 μg/mL (P < 0.01; Fig 7), whereas responses decreased during the second to sixth dark intervals when 10 μg/mL CBD was administered to larvae pre-treated with 0.5 μg/mL WIN (P < 0.01; Fig 7). In contrast to WIN0.5 + CBD1 (μg/mL), responses decreased during the fifth and sixth dark intervals when 10 μg/mL CBD was administered to larvae pre-treated with 1 μg/mL WIN (P < 0.01; Fig 8). The combined effects of CBD and WIN (0.5 and 1 μg/mL) indicated that CBD and WIN administered in a 1:1 ratio induced recovery (Figs 7 and 8). CBD5 + WIN0.5 (μg/mL), CBD0.5 + WIN1 (μg/mL), and CBD5 + WIN1 (μg/mL) tended to weaken responses during the first dark interval, and significant attenuation was observed in larvae exposed to CBD5 + WIN1 (μg/mL) (P < 0.05; Figs 7 and 8).

## Discussion

In this study, we analyzed the abnormal behavior of zebrafish exposed to the cannabinoids cannabidiol and WIN55,212–2, Using these two cannabinoids, we investigated the effects in the acute phase, withdrawal phase, and to their combined use. The release of serotonin mediated by the activation of cannabinoid receptors may also regulate noradrenergic and dopaminergic neurotransmission because cannabinoids (WIN) modulate motor responses by activating dopaminergic and glutamatergic neurons [33]. Psychoactive drugs, such as WIN and THC, that activate cannabinoid receptors cause hypothermia and hypoactivity (including catalepsy-like immobilization), which are the inverse of the symptoms associated with the serotonin syndrome [34, 35].

Even at a low WIN concentration (0.5 μg/mL), we observed reductions in the ambulatory activity and responses of zebrafish larvae. Reportedly, activation of 5-HT1A receptors in response to CBD attenuates distinct drug-induced catalepsy in mice [29, 36].

Some studies have revealed that CBD or 5-HT receptor agonists have adverse effects such as antiepileptic seizure [37], whereas other studies have revealed the developmental effects of CBD and THC in zebrafish; however, further studies are needed to assess the latent effects [38].

Given that the toxic effects of WIN are considerably more pronounced than those of CBD, we should recognize that in cases of sudden death associated with cannabinoids, drugs such as WIN are likely to promote cardiopulmonary dysfunction. We found that both CBD and WIN (even at a low dose) attenuated locomotor activity and responses in zebrafish larvae, with WIN showing a stronger principal toxic action than CBD, whereas high concentration of CBD notably attenuated intense hyperactivity in the dark. However, after a 24-h withdrawal period, we observed recovery in both pre-treated groups. We also demonstrated that interaction between WIN and CBD can enhance locomotory activity and even facilitated a comparative recovery. Reactions tended to differ with different combinations of different concentrations of CBD and WIN, with combinations comprising considerably higher concentrations of CBD inducing hyperactivity during light periods.

In the repeated light and dark test, responses the first and second intervals may be somewhat inconsistent, whereas the results from the third interval and thereafter are likely to be more representative of the actual state. Therefore, further studies are needed to confirm the behavioral analysis method and responses in zebrafish.

In recent years, there has been an increase in the number of studies that have examined the pharmacological effects of cannabinoids using zebrafish as model animals, which is reflective of the fact that cannabinoids (such as CBD) are increasingly being used for medicinal purposes worldwide [37, 39]. Our study augments other research on cannabinoid biology, cannabinoid 1 and 2 receptors, and 5-HT1A function in zebrafish [32, 40, 41]. Locomotory activity in response to light stimuli using zebrafish has been previously evaluated [16, 25, 32, 42], and in the present study, we found that exposure to light attenuated activity, whereas darkness tended to induce activity in normal zebrafish.

In summary, the repeated light and dark test is an appropriate method for evaluating the responses of zebrafish to variations in their surrounding environment. We found that CBD and WIN induced temporary locomotive disorders and that drug withdrawal for 24 h resulted in an attenuation of drug-induced low activity. On the basis of these observations, we can conclude that assessing symptoms during and after drug exposure is a valid method for investigating pharmacological effects in a fish model, and we believe that our findings have important implications with respect to the persistence of drug-associated complications.

Future studies using zebrafish models should determine whether CBD can attenuate other cannabinoid-induced abnormal behaviors or side effects attributable to psychomimetic drugs, including antipsychotic agents. Although several studies have attempted to duplicate and assess cannabinoid-induced motor functional disorders in both zebrafish and rodents, these have had little success in accurately replicating the results obtained using rodent models. Thus, further research is necessary to evaluate toxic effects and/or drug-induced abnormal behavior in zebrafish. Our results will, nevertheless. contribute to elucidating the potential links between cannabinoids and unexpected abnormal behaviors.

## Supporting information

S1 File (RTF)

S2 File (RTF)

S3 File (RTF)

S4 File (RTF)

S5 File (RTF)

S6 File (RTF)
