# Supplementary material for: Analyzing cannabinoid-induced abnormal behavior in a zebrafish model
Source: PLoS One. 2020 Oct 8;15(10):e0236606. doi: 10.1371/journal.pone.0236606 (PMC7544081; doi:10.1371/journal.pone.0236606)
Supplement: S1 File — (RTF) [file pone.0236606.s001.rtf]

1.	CBD (Total distance) (mm)

Control�@�@�@�@ MET�@�@�@�@�@ CBD0.5 �@�@   CBD1  �@�@   CBD5  �@�@   CBD10
Mean	10247.72	11129.69	8490.591	9331.751	9850.076	8255.247	
SEM	690.4832	580.0485	1019.224	718.8323	646.8486	728.1092	


2.	CBD (Moving distance for light or dark stimulation) (mm)

Control
         �@�@�@�@�@ 1st. ON  �@     1st. OFF �@    2nd. ON �@    2nd. OFF �@   3rd. ON  �@   3rd. OFF �@   4th. ON  �@�@   4th. OFF �@    5th. ON �@�@   5th. OFF �@    6th. ON �@    6th. OFF  
Mean	906.251625	1204.809	779.6034	1243.693	723.5549	1230.035	627.5537	1177.055	625.0027	1182.531	679.4737	1045.151	
SEM	98.8271699	118.6195	96.6812	130.075	104.4919	138.2268	68.79061	128.9733	83.89647	148.5726	94.39268	114.3732	

MET
          �@�@�@�@�@1st. ON  �@      1st. OFF �@    2nd. ON �@     2nd. OFF�@�@    3rd. ON �@     3rd. OFF�@�@    4th. ON �@     4th. OFF �@    5th. ON �@�@�@   5th. OFF      6th. ON �@�@    6th. OFF  
Mean	810.05924	1288.408	833.6209	1171.83	647.0513	1244.587	699.6826	1186.368	700.8286	1126.379	613.2989	1117.028	
SEM	58.8379176	145.4741	98.53116	119.743	36.86473	140.1781	37.06749	130.2112	47.66491	128.4112	49.73988	 106.6659	

CBD0.5 (ìg/mL)
        �@�@�@�@�@  1st. ON�@        1st. OFF �@�@    2nd. ON      2nd. OFF �@�@   3rd. ON �@     3rd. OFF�@�@    4th. ON�@      4th. OFF�@     5th. ON�@�@�@    5th. OFF      6th. ON�@�@     6th. OFF  
Mean	518.840857	929.9798	701.7866	851.264	476.9785	852.0131	481.5287	805.9281	463.7403	759.9664	492.015	768.2179	
SEM	49.0302938	134.2814	89.08546	136.0536	55.05084	134.5918	54.43235	141.9582	64.07971	144.6903	59.77358	139.0964	

CBD1 (ìg/mL)
         �@�@�@�@�@ 1st. ON       1st. OFF�@�@     2nd. ON      2nd. OFF    3rd. ON �@�@    3rd. OFF     4th. ON �@     4th. OFF     5th. ON �@�@   5th. OFF      6th. ON�@     6th. OFF  
Mean	716.301125	1025.765	750.2384	952.0431	643.0075	865.4651	631.8303	884.3618	441.0717	872.8231	513.5638	901.2386	
SEM	116.51075	110.8628	101.397	128.628	61.04078	142.3166	59.90164	150.4103	55.99405	101.1405	93.22018	98.01653	

CBD5 (ìg/mL)
         �@�@�@ 1st. ON �@�@      1st. OFF�@     2nd. ON�@     2nd. OFF�@    3rd. ON�@�@     3rd. OFF     4th. ON �@�@    4th. OFF�@     5th. ON�@�@    5th. OFF�@    6th. ON  �@�@    6th. OFF 
Mean	1073.42513	1239.248	804.3336	1123.914	540.3551	864.957	604.2035	917.8881	646.174	850.1795	646.7126	918.5717	
SEM	135.521068	116.7144	110.9315	95.36771	65.61747	49.37933	87.85024	85.38926	115.3323	63.69837	106.9988	87.8164	

CBD10 (ìg/mL)
          �@�@�@�@ 1st. ON�@       1st. OFF �@�@   2nd. ON �@    2nd. OFF �@  3rd. ON �@     3rd. OFF �@   4th. ON �@    4th. OFF �@    5th. ON  �@�@  5th. OFF�@     6th. ON  �@   6th. OFF 
Mean	904.24375	1033.77	974.596	779.009	788.5408	602.3382	716.8528	 653.0831	713.4271	566.6301	563.2527	441.8613	
SEM	165.603468	122.6913	116.2059	137.1428	90.00869	117.493	97.18923	113.9404	100.8526	76.61839	62.83953	73.39462	

 


3.	CBD (Velocity in dark) (mm/s)

Control�@�@�@�@�@ MET�@�@�@ �@�@CBD0.5  �@�@  CBD1  �@�@   CBD5  �@�@   CBD10
Mean
1.3311743
1.242774
0.9227267
1.0219821
1.1070561
0.7573125

SEM
0.140372

0.119227

0.145813

0.117947

0.078105

0.105431


4.	CBD (Moving Duration) (sec)

Control�@�@�@�@�@ MET�@�@�@�@ CBD0.5  �@�@  CBD1  �@�@   CBD5 �@�@�@    CBD10
Mean
2558.073

2457.41

1727.386

2153.901

2261.333

1832.48


		
SEM
166.69723

181.78967

240.64887

205.46368

169.14343

205.70294


		
