# Supplementary material for: Analyzing cannabinoid-induced abnormal behavior in a zebrafish model
Source: PLoS One. 2020 Oct 8;15(10):e0236606. doi: 10.1371/journal.pone.0236606 (PMC7544081; doi:10.1371/journal.pone.0236606)
Supplement: S2 File — (RTF) [file pone.0236606.s002.rtf]

1.	CBD+WIN0.5 (Total distance) (mm)

Control�@�@ �@�@�@M+D�@�@�@�@ C0.5+W0.5  �@C1+W0.5 �@�@ C5+W0.5  �@ C10+W0.5
Mean	5584.296	5744.47	6279.846	7542.239	5494.78	6295.605	
SEM	427.2108	267.5132	397.6968	780.0112	322.8075	474.9327	


2.	CBD+WIN0.5 (Moving distance for light or dark stimulation) (mm)

Control
      �@�@�@�@     1st. ON �@    1st. OFF  �@�@  2nd. ON  �@   2nd. OFF �@   3rd. ON�@     3rd. OFF�@�@    4th. ON �@    4th. OFF �@    5th. ON�@�@�@    5th. OFF �@   6th. ON �@   6th. OFF 
Mean	164.7687	826.6558	159.7294	870.9465	120.7085	751.6474	123.9705	775.9265	185.4545	705.24	127.0974	801.2689	
SEM	15.99314	126.2063	7.534462	100.871	15.2362	91.37674	18.59873	92.49766	9.072113	66.30221	17.466	85.97729	

MET+DMSO(M+D)
      �@�@�@�@     1st. ON �@     1st. OFF  �@   2nd. ON   �@  2nd. OFF �@   3rd. ON  �@�@   3rd. OFF �@   4th. ON   �@�@  4th. OFF �@   5th. ON �@�@   5th. OFF �@�@   6th. ON  �@    6th. OFF
 Mean	156.2325	776.46	182.9945	801.4198	169.2368	787.0808	175.4916	769.0543	207.3658	758.1753	196.9109	775.4846	
SEM	17.36737	36.32636	18.63902	49.48318	23.60959	83.03355	20.92388	55.82302	18.07962	51.04991	16.8827	43.6969	

CBD0.5+WIN0.5(C0.5+W0.5) (ìg/mL)
    �@�@�@�@       1st. ON�@      1st. OFF     2nd. ON  �@�@   2nd. OFF �@   3rd. ON�@     3rd. OFF  �@  4th. ON  �@�@�@   4th. OFF     5th. ON �@   5th. OFF�@�@    6th. ON  �@   6th. OFF
Mean	195.5001	769.2249	170.9809	848.5449	159.0676	857.8546	176.5333	900.36	189.3406	976.8047	163.8014	1053.819	
SEM	24.38289	130.4233	23.1738	93.99144	25.87101	56.35767	20.9886	82.951	29.52904	94.63088	18.97003	96.49492	

CBD1+WIN0.5(C1+W0.5) (ìg/mL)
    �@�@�@      1st. ON  �@    1st. OFF�@    2nd. ON  �@�@   2nd. OFF    3rd. ON  �@�@   3rd. OFF�@    4th. ON  �@   4th. OFF �@    5th. ON �@�@   5th. OFF �@�@   6th. ON �@    6th. OFF 
Mean	126.7286	893.5423	183.6051	1071.93	156.2657	1058.139	113.9036	1260.633	156.4777	1262.484	160.9586	1151.949	
SEM	12.80518	150.5563	29.21028	150.073	30.50251	141.1608	13.50676	140.8801	24.33934	151.6962	21.19409	135.5577	

CBD5+WIN0.5(C5+W0.5) (ìg/mL)
       �@�@�@   1st. ON  �@    1st. OFF �@   2nd. ON  �@   2nd. OFF  �@  3rd. ON   �@�@  3rd. OFF �@�@   4th. ON  �@  4th. OFF �@�@   5th. ON�@     5th. OFF�@�@    6th. ON �@    6th. OFF
Mean	132.5184	405.3184	206.9304	727.3802	130.3193	875.0878	152.293	1015.013	170.7436	1087.131	184.2718	1132.131	
SEM	13.67236	57.09339	30.76311	130.3448	15.05242	127.286	27.26548	117.3099	21.79706	111.3614	21.8747	129.0925	

CBD10+WIN0.5(C10+W0.5) (ìg/mL)
       �@�@�@     1st. ON �@    1st. OFF �@�@   2nd. ON �@    2nd. OFF �@�@   3rd. ON �@    3rd. OFF  �@  4th. ON �@    4th. OFF �@    5th. ON �@�@   5th. OFF �@    6th. ON  �@   6th. OFF 
Mean	239.1704	912.9164	428.3474	822.3656	467.16	555.8195	306.3916	642.8465	331.8534	658.5259	356.0346	644.3408	
SEM	24.04576	116.7505	59.07167	128.2598	65.86034	107.3964	45.41744	81.82028	56.45369	101.9553	51.68482	118.0715	


3.	CBD+WIN0.5 (Velocity in dark) (mm/s) 

Control�@�@ �@�@�@M+D�@�@�@�@ C0.5+W0.5 �@�@ C1+W0.5  �@�@C5+W0.5  �@ C10+W0.5
Mean
0.876232
1.043779
0.973348
1.240491
0.819339
0.802222

SEM
0.082012
0.127297
0.074496
0.14749
0.05547
0.101558


4.	CBD+WIN0.5 (Moving Duration) (sec)

Control�@�@�@�@�@M+D�@�@�@�@�@ C0.5+W0.5 �@ C1+W0.5   �@�@C5+W0.5  �@ C10+W0.5
Mean
1217.709
1410.921
1331.519
1776.585
1448.193
1425.622

SEM
142.1302
176.2438
106.11
233.2521
165.6339
132.0607


		929.9798	701.7866	851.264	476.9785	852.0131	481.5287	805.9281	463.7403	759.9664	492.015	768.2179	
		134.2814	89.08546	136.0536	55.05084	134.5918	54.43235	141.9582	64.07971	144.6903	59.77358	139.0964	
