# Supplementary material for: Analyzing cannabinoid-induced abnormal behavior in a zebrafish model
Source: PLoS One. 2020 Oct 8;15(10):e0236606. doi: 10.1371/journal.pone.0236606 (PMC7544081; doi:10.1371/journal.pone.0236606)
Supplement: S3 File — (RTF) [file pone.0236606.s003.rtf]

1.	CBD+WIN1 (Total distance) (mm)

Control    �@�@ M+D�@   �@ C0.5+W1      C1+W1       C5+W1      C10+W1
Mean	5794.216	5933.679	5956.76	6573.978	5555.25	4588.909	
SEM	526.3623	375.46	702.6346	719.6552	380.1843	499.3695	


2.	CBD+WIN1 (Moving distance for light or dark stimulation) (mm)

Control
                1st. ON       1st. OFF      2nd. ON      2nd. OFF       3rd. ON      3rd. OFF      4th. ON      4th. OFF      5th. ON      5th. OFF       6th. ON      6th. OFF
Mean	103.7879	783.142	57.86248	908.1191	105.6017	851.5749	80.12123	1008.338	86.67454	1044.603	62.46464	1038.452	
SEM	16.83439	53.53018	8.350435	69.51964	10.52596	120.6584	13.10614	97.9057	14.40054	96.50525	7.888487	93.38576	

MET+DMSO(M+D)
              1st. ON       1st. OFF      2nd. ON      2nd. OFF      3rd. ON       3rd. OFF      4th. ON      4th. OFF      5th. ON      5th. OFF       6th. ON      6th. OFF
Mean	93.18894	867.8462	80.66102	920.9638	81.90219	828.8328	97.46232	998.3657	78.8905	964.021	85.89906	973.0548	
SEM	11.98291	35.00394	10.19565	69.22287	10.76128	129.9078	12.70337	106.5107	3.992255	116.1547	11.22919	104.6459	

CBD0.5+WIN1(C0.5+W1) (ìg/mL)
              1st. ON       1st. OFF      2nd. ON      2nd. OFF      3rd. ON       3rd. OFF      4th. ON      4th. OFF      5th. ON       5th. OFF      6th. ON      6th. OFF
Mean	95.19163	459.0767	83.19565	985.5935	100.5065	893.6457	72.96272	1086.036	110.7787	1051.175	75.85191	922.9064	
SEM	13.19908	113.6292	13.62123	176.6373	10.97067	130.912	9.178818	159.555	11.34772	144.6419	8.490996	144.2782	

CBD1+WIN1(C1+W1) (ìg/mL)
                1st. ON       1st. OFF      2nd. ON      2nd. OFF     3rd. ON       3rd. OFF      4th. ON       4th. OFF      5th. ON       5th. OFF      6th. ON      6th. OFF
Mean	74.23165	904.7331	101.9093	1088.03	91.75017	1008.212	79.32158	1045.219	110.0952	1159.982	96.05496	1136.377	
SEM	10.48638	243.6765	15.8118	167.5875	11.50377	144.3458	7.376519	148.9495	15.51871	142.3365	10.67345	150.3492	

CBD5+WIN1(C5+W1) (ìg/mL)
              1st. ON       1st. OFF      2nd. ON      2nd. OFF      3rd. ON      3rd. OFF      4th. ON       4th. OFF      5th. ON       5th. OFF      6th. ON      6th. OFF
Mean	130.7563	467.5744	126.5973	854.2156	113.5421	994.0604	104.4261	923.3699	142.3681	933.9901	201.0374	728.6171	
SEM	18.46721	75.52652	20.8127	100.7016	13.995	120.2167	6.199397	125.6037	18.38946	85.68397	36.85984	79.73018	

CBD10+WIN1(C10+W1) (ìg/mL)
                1st. ON       1st. OFF      2nd. ON      2nd. OFF      3rd. ON       3rd. OFF     4th. ON       4th. OFF      5th. ON       5th. OFF      6th. ON      6th. OFF
  Mean	104.5915	796.2889	116.6142	882.1479	116.2436	758.906	85.32543	661.857	94.69516	522.4473	93.62089	448.8712	
SEM	9.652146	176.4324	14.79901	139.2143	14.82414	136.7309	14.39726	99.58075	9.312417	82.64165	15.25278	89.71108	


3.	CBD+WIN1 (Velocity in dark) (mm/s) 

Control�@�@    M+D�@�@�@    C0.5+W1     C1+W1       C5+W1      C10+W1
Mean
1.073257
0.798999
1.0024
1.125581
0.962657
0.748309

SEM
0.11078
0.062775
0.132923
0.130768
0.090712
0.092208


4.	CBD+WIN1 (Moving Duration) (sec)

Control�@�@    M+D�@�@  �@  C0.5+W1      C1+W1      C5+W1      C10+W1
Mean
1517.615
951.2449
1336.419
1504.39
1369.788
955.6532

SEM
188.0255
94.95824
169.7942
216.69
181.0763
123.0788


		929.9798	701.7866	851.264	476.9785	852.0131	481.5287	805.9281	463.7403	759.9664	492.015	768.2179	
		134.2814	89.08546	136.0536	55.05084	134.5918	54.43235	141.9582	64.07971	144.6903	59.77358	139.0964	
