# Supplementary material for: Analyzing cannabinoid-induced abnormal behavior in a zebrafish model
Source: PLoS One. 2020 Oct 8;15(10):e0236606. doi: 10.1371/journal.pone.0236606 (PMC7544081; doi:10.1371/journal.pone.0236606)
Supplement: S4 File — (RTF) [file pone.0236606.s004.rtf]

1.	WIN (Total distance) (mm)

Control�@�@�@  DMSO�@�@�@    WIN0.5      WIN1        WIN5       WIN10
Mean	9497.322	10274.81	3266.928	4216.006	456.3698	207.7894	
SEM	424.5442	589.4983	382.3852	495.0124	56.93991	44.61808	


2.	WIN (Moving distance for light or dark stimulation) (mm)

Control
              1st. ON       1st. OFF      2nd. ON       2nd. OFF      3rd. ON      3rd. OFF      4th. ON       4th. OFF      5th. ON      5th. OFF      6th. ON       6th. OFF 
 Mean	645.8135	1154.353	733.2439	1183.881	629.634	1105.681	627.8327	1056.004	503.2862	971.0464	442.2935	799.773	
SEM	90.76617	56.84564	98.55084	64.44779	76.05085	79.41408	76.96742	62.05625	44.46739	57.28605	39.98882	50.77275	

DMSO
              1st. ON       1st. OFF      2nd. ON       2nd. OFF     3rd. ON      3rd. OFF       4th. ON       4th. OFF     5th. ON       5th. OFF      6th. ON       6th. OFF  
Mean	802.4763	1120.243	681.2098	1204.316	702.8743	1031.835	561.9099	1065.156	628.8749	1080.829	568.2019	826.8804	
SEM	132.0752	86.26484	67.6273	66.79623	44.07293	77.97206	55.45187	72.20668	59.11976	72.52653	50.96677	87.26823	

WIN0.5(ìg/mL)
              1st. ON       1st. OFF      2nd. ON       2nd. OFF     3rd. ON       3rd. OFF      4th. ON       4th. OFF     5th. ON       5th. OFF      6th. ON       6th. OFF
 Mean	259.0545	447.8896	269.8179	304.4598	257.4172	393.1118	282.5842	260.4166	274.3915	210.8874	234.1322	179.2568	
SEM	31.6226	81.45964	48.85565	50.90156	22.95071	48.61883	51.94439	44.67247	43.49672	35.53235	44.8522	28.73431	

WIN1(ìg/mL)
              1st. ON       1st. OFF      2nd. ON       2nd. OFF     3rd. ON       3rd. OFF      4th. ON       4th. OFF     5th. ON       5th. OFF      6th. ON       6th. OFF 
 Mean	188.0572	238.0317	212.3505	454.7395	294.9732	571.0129	227.6995	452.849	368.2121	490.7855	390.2432	524.3316	
SEM	22.13019	35.48899	19.41484	100.6107	39.55503	92.43253	35.9217	89.35486	75.39424	85.21591	71.76959	98.23026	

WIN5(ìg/mL)
              1st. ON       1st. OFF      2nd. ON       2nd. OFF     3rd. ON       3rd. OFF      4th. ON      4th. OFF       5th. ON      5th. OFF      6th. ON       6th. OFF
 Mean	137.1988	234.1444	59.06866	25.57847	10.74598	8.816749	8.666929	5.024125	8.116834	12.23686	10.28111	7.176913	
SEM	26.27067	49.60614	9.548419	5.273545	1.800981	1.435865	1.279701	0.884193	1.563995	2.285168	1.675914	1.217178	

WIN10(ìg/mL)
              1st. ON       1st. OFF      2nd. ON       2nd. OFF     3rd. ON       3rd. OFF      4th. ON       4th. OFF      5th. ON      5th. OFF      6th. ON       6th. OFF
 Mean	30.93096	14.86737	33.69148	46.68021	45.90706	36.38056	43.66737	28.03861	10.50794	4.659041	20.89789	11.3747	
SEM	5.503855	2.366621	4.508141	8.369831	7.684423	7.745751	9.041748	8.024577	1.930373	0.848223	5.508857	2.226556	


3.	WIN (Velocity in dark) (mm/s)

Control�@�@�@   DMSO�@�@�@   WIN0.5     WIN1        WIN5        WIN10
Mean
0.768383
0.70336
0.34379
0.540729
0.064155
0.028002

SEM
0.10779
0.089253
0.041045
0.084151
0.012595
0.008608


4.	WIN (Moving Duration) (sec)

Control�@�@�@   DMSO�@�@�@   WIN0.5      WIN1        WIN5        WIN10
Mean
1739.697
1578.688
574.5459
854.4441
99.46008
3.661385

SEM
295.2415
213.1886
80.33835
157.6468
26.87696
1.763206


		929.9798	701.7866	851.264	476.9785	852.0131	481.5287	805.9281	463.7403	759.9664	492.015	768.2179	
		134.2814	89.08546	136.0536	55.05084	134.5918	54.43235	141.9582	64.07971	144.6903	59.77358	139.0964	
