# Supplementary material for: Analyzing cannabinoid-induced abnormal behavior in a zebrafish model
Source: PLoS One. 2020 Oct 8;15(10):e0236606. doi: 10.1371/journal.pone.0236606 (PMC7544081; doi:10.1371/journal.pone.0236606)
Supplement: S5 File — (RTF) [file pone.0236606.s005.rtf]

1.	Withdrawal of CBD (Total distance) (mm)

Control�@�@�@   MET�@   �@�@ CBD0.5      CBD1        CBD5        CBD10
Mean	6495.683	6821.593	5618.056	6943.461	8006.699	8140.071	
SEM	315.4814	471.4702	501.1844	123.6462	554.1142	424.4627	

							
2.	Withdrawal of CBD (Moving distance for light or dark stimulation) (mm)

Control
                1st. ON       1st. OFF      2nd. ON       2nd. OFF     3rd. ON      3rd. OFF       4th. ON      4th. OFF       5th. ON      5th. OFF      6th. ON       6th. OFF  
Mean	424.1379	908.1423	377.2482	1057.38	319.1911	822.0359	238.82	712.2968	256.417	671.7602	206.3249	701.8985	
SEM	37.56498	43.3406	54.49351	39.33862	46.08634	67.62434	39.20627	67.35698	29.48565	56.3409	25.24816	41.7949	


MET
               1st. ON        1st. OFF      2nd. ON      2nd. OFF      3rd. ON      3rd. OFF      4th. ON       4th. OFF      5th. ON       5th. OFF      6th. ON       6th. OFF 
 Mean	451.8529 	934.5538	381.9109	895.8514	399.6319	849.3909	291.4487	752.7468	317.3027	780.6538	269.6489	692.3955	
SEM	75.92323	71.85914	42.37991	62.98197	55.71987	72.80444	35.71075	75.29813	51.15197	67.29961	50.51091	70.44668	

CBD0.5(ìg/mL)
                1st. ON       1st. OFF      2nd. ON       2nd. OFF     3rd. ON      3rd. OFF       4th. ON       4th. OFF     5th. ON       5th. OFF      6th. ON       6th. OFF  
Mean	300.0604	798.2124	264.3033	759.8084	278.4085	675.4488	277.0005	649.5074	294.5104	575.0109	268.2864	546.4741	
SEM	40.09167	89.88235	30.29028	102.781	34.84371	90.97993	32.87972	93.85937	34.34839	75.61731	34.14813	56.50901	

CBD1(ìg/mL)
               1st. ON        1st. OFF      2nd. ON       2nd. OFF     3rd. ON       3rd. OFF      4th. ON      4th. OFF      5th. ON       5th. OFF      6th. ON       6th. OFF 
Mean	230.7767	912.1861	 298.3742	1004.311	317.3696	806.2128	324.9894	754.3409	258.4344	717.0067	292.7649	683.452	
SEM	25.39447	91.0327	40.44146	67.91313	59.56085	48.78324	53.3482	33.97619	38.96828	70.10556	42.08942	57.97957	


CBD5(ìg/mL)
                1st. ON       1st. OFF      2nd. ON       2nd. OFF     3rd. ON        3rd. OFF     4th. ON      4th. OFF      5th. ON       5th. OFF      6th. ON       6th. OFF
 Mean	314.7565	1115.365	297.9785	1194.574	346.3553	1111.93	344.7775	1023.611	265.8166	900.2443	190.0546	931.7386	
SEM	48.80663	90.10124	52.70137	114.9076	46.36007	100.8525	57.04414	107.0198	39.51485	80.02739	28.02263	83.87802	

CBD10(ìg/mL)
                1st. ON       1st. OFF      2nd. ON       2nd. OFF     3rd. ON       3rd. OFF      4th. ON      4th. OFF      5th. ON       5th. OFF      6th. ON       6th. OFF  
Mean	328.982	1132.752	556.6979	1201.587	396.9873	1038.191	318.2459	906.6457	322.7813	844.3736	265.3102	912.6708	
SEM	37.98663	71.66256	83.75026	77.99388	47.24359	76.76478	36.96037	77.33847	37.78091	88.71077	31.22948	104.8424	


3.	Withdrawal of CBD (Velocity in dark) (mm/s)

Control�@�@�@   MET�@�@�@     CBD0.5      CBD1        CBD5        CBD10
Mean
0.926742
0.923777

0.735021

0.951569

1.162521

1.117844


SEM    
0.048279
0.078468
0.08698
0.025036
0.096627
0.075886


4.	Withdrawal of CBD (Moving Duration) (sec)

Control�@�@�@   MET�@�@�@    CBD0.5       CBD1        CBD5        CBD10
Mean
1467.893

1548.629

1220.663

1469.616

1872.485

1896.652


		929.9798	701.7866	851.264	476.9785	852.0131	481.5287	805.9281	463.7403	759.9664	492.015	768.2179	
SEM
80.37766

141.8226

134.4491

92.45131

160.8188

136.1107


		134.2814	89.08546	136.0536	55.05084	134.5918	54.43235	141.9582	64.07971	144.6903	59.77358	139.0964	
