# Supplementary material for: Analyzing cannabinoid-induced abnormal behavior in a zebrafish model
Source: PLoS One. 2020 Oct 8;15(10):e0236606. doi: 10.1371/journal.pone.0236606 (PMC7544081; doi:10.1371/journal.pone.0236606)
Supplement: S6 File — (RTF) [file pone.0236606.s006.rtf]

1.	Withdrawal of WIN (Total distance) (mm)

Control�@�@�@  DMSO�@�@�@   WIN0.5      WIN1
Mean	6360.266	6540.863	6135.211	4763.563	
SEM	380.8104	168.675	585.6944	275.842	


2.	Withdrawal of WIN (Moving distance for light or dark stimulation) (mm)

Control
              �@ 1st. ON       1st. OFF      2nd. ON      2nd. OFF      3rd. ON       3rd. OFF      4th. ON       4th. OFF     5th. ON       5th. OFF      6th. ON       6th. OFF
Mean	298.4605	1019.002	329.5998	939.3933	316.1734	862.8839	305.6418	772.8264	225.5383	641.4216	226.8041	470.5865		
SEM	36.85363	62.25479	57.6025	41.00929	39.40541	58.88215	43.11421	71.79671	35.11725	63.89098	30.13792	61.824		
 
DMSO
              �@�@ 1st. ON       1st. OFF      2nd. ON       2nd. OFF     3rd. ON       3rd. OFF      4th. ON      4th. OFF      5th. ON       5th. OFF      6th. ON      6th. OFF 
Mean	294.1138	1109.658	306.1375	1011.595	270.0697	938.9458	224.3358	802.4853	236.1122	589.5224	210.8254	589.0164	
SEM	18.82516	49.2613	11.2677	53.39202	23.18514	45.50521	22.24841	26.51085	15.35131	23.70644	17.95457	32.00904	

WIN0.5(ìg/mL)
        �@    �@  1st. ON      1st. OFF      2nd. ON       2nd. OFF     3rd. ON       3rd. OFF      4th. ON      4th. OFF      5th. ON       5th. OFF      6th. ON       6th. OFF
Mean	174.74	709.5466	265.2114	925.699	257.8609	921.4364	178.3897	934.8427	209.7291	823.2147	228.2351	772.5736			
SEM	33.73979	99.64965	53.79423	109.2684	33.5652	110.1948	23.13547	151.9849	19.73615	122.5102	33.03353	101.4745			
 
WIN1(ìg/mL)
      �@�@         1st. ON       1st. OFF      2nd. ON      2nd. OFF      3rd. ON       3rd. OFF      4th. ON      4th. OFF      5th. ON       5th. OFF      6th. ON      6th. OFF
  Mean	192.4646	607.4601	269.7561	698.3274	236.8982	659.7229	172.6057	595.5337	170.9769	495.285	254.3182	443.7168	
SEM	18.71248	40.42503	43.17402	60.09493	33.85136	57.04448	20.33777	50.87169	23.37906	44.60389	27.23533	37.50615	


3.	Withdrawal of WIN (Velocity in dark) (mm/s)

Control�@�@�@   DMSO�@�@�@   WIN0.5       WIN1 
Mean
0.576182
0.550466
0.910005
0.621466

SEM
0.04858
0.050243
0.109933
0.039906

    

4.	Withdrawal of WIN (Moving Duration) (sec)

Control�@�@�@   DMSO�@�@�@   WIN0.5       WIN1 
Mean
804.6031
746.1065
1580.415
852.202

SEM
82.54513
67.14092
253.7093
53.31926

   


		929.9798	701.7866	851.264	476.9785	852.0131	481.5287	805.9281	463.7403	759.9664	492.015	768.2179	
		134.2814	89.08546	136.0536	55.05084	134.5918	54.43235	141.9582	64.07971	144.6903	59.77358	139.0964	
